# ‘Tuberculosis pericarditis’: a case report in a high-income country

**DOI:** 10.1093/ehjcr/ytaf050

**Published:** 2025-01-31

**Authors:** Carolina Miguel Gonçalves, Margarida Cabral, Rita Martins, Maria João Silva, Hélia Martins

**Affiliations:** Department of Cardiology, Leiria Local Health Unit, Rua das Olhalvas, Leiria 2410-197, Portugal; Department of Cardiology, Leiria Local Health Unit, Rua das Olhalvas, Leiria 2410-197, Portugal; Department of Pneumology, Leiria Local Health Unit, Rua das Olhalvas, Leiria 2410-197, Portugal; Department of Pneumology, Leiria Local Health Unit, Rua das Olhalvas, Leiria 2410-197, Portugal; Department of Cardiology, Leiria Local Health Unit, Rua das Olhalvas, Leiria 2410-197, Portugal

**Keywords:** Cardiac tamponade, Swinging heart, *Mycobacterium tuberculosis*, Pericardial tuberculosis, Pulmonary tuberculosis, Case report

## Abstract

**Background:**

The incidence of tuberculous (TB) infection varies greatly geographically. In endemic countries, it is one of the major aetiologies of pericardial diseases, whereas it is an uncommon cause in industrialized countries. The mortality rate of TB pericarditis complications is up to 40%, emphasizing the importance of early diagnosis and management.

**Case summary:**

An 82-year-old woman presented with fever, dry cough, and constitutional symptoms for 2 weeks. The electrocardiogram showed low-voltage complexes, chest X-ray showed unspecific changes, and blood work revealed mild anaemia and a slight elevation of inflammatory parameters. A diagnosis of pulmonary infection was assumed, and the patient was discharged with antibiotics. One month later, she presented with worsening exertion fatigue and an increase in cardiothoracic index was noted on the chest X-ray. Further imaging studies by computed tomography and echocardiography revealed a severe pericardial effusion (PE) with echocardiographic signs of hemodynamic instability. The patient underwent a pericardiocentesis. Polymerase chain reaction study for *Mycobacterium tuberculosis* in the pericardial fluid was positive. Pulmonary involvement was confirmed by videobronchoscopy with bronchoalveolar lavage. The patient received tetraconjugate management and corticosteroids with an adequate clinical response. The follow-up echocardiographic assessment showed mild PE with no constrictive physiology.

**Discussion:**

This is a case of definitive TB pericarditis that emphasizes the potential increase in TB cases in non-endemic countries and the need for a high clinical suspicion ensure early diagnosis and treatment, reducing complications and mortality of this disease.

**Learning points**

- In cases of non-specific and constitutional symptoms associated with pericardial effusion, particularly in high-risk populations, diagnosis of tuberculous (TB) pericarditis should be considered.
- Pericardiocentesis for both diagnosis and treatment, antituberculosis drugs, and corticosteroids in selected cases are recommended to control the infection and prevent complications.
- Clinicians should be aware of a potential increase in TB pericarditis in non-endemic countries to enable earlier diagnosis and management.

## Introduction

Globally, tuberculosis (TB) is the most common infectious cause of death.^1,2^ The incidence of TB varies by region.^1^ According to the World Health Organization, Portugal falls into the lower–moderate incidence category.^3^

Although primarily a pulmonary disease, ∼60% of TB patients have cardiovascular involvement, with the pericardium, myocardium, and aorta being the most affected structures.^4^ Pericardial involvement includes pericardial effusion (PE), effusive-constrictive pericarditis, and constrictive pericarditis (CP).^5,6^ Tuberculosis accounts for <4% of pericardial diseases in high-income countries^5^ and is considered rare in Europe.^6^

On the other hand, TB is a common cause of significant PE in endemic countries, accounting for more than 90% of cases in human immunodeficiency virus (HIV)-infected patients and 50–70% in non-HIV patients.^5^ Accordingly, it is the most prevalent cause of CP in low- and middle-income countries, in contrast to idiopathic or viral aetiologies in high-income countries.^7^

In cases where the clinical presentation suggests TB pericarditis—such as in patients with fever, chest pain, dyspnoea, and risk factors such as immunosuppression or prior TB exposure—pericardiocentesis should be considered.^5^ According to the European Society of Cardiology guidelines, the presence of tubercle bacilli in the pericardial fluid or on histological section of the pericardium [culture or polymerase chain reaction (PCR)] is necessary for a definite diagnosis of TB pericarditis.^5^ Moreover, screening of pulmonary TB is required, as it coexists in 30% of the patients.^6^

Addressing TB pericarditis requires a multidisciplinary team,^6^ involving a 6-month regimen of antituberculosis drugs—at least 2 months of rifampicin, isoniazid, pyrazinamide, and ethambutol followed by rifampicin and isoniazid.^5^

Cardiac tamponade and pericardial constriction are potential complications,^5,7^ with an associated mortality rate of 17–40% within 6 months after diagnosis.^5^ There is the need to improve diagnosis, develop better treatments, and reduce morbidity and mortality.^8^

## Summary figure

**Figure:**
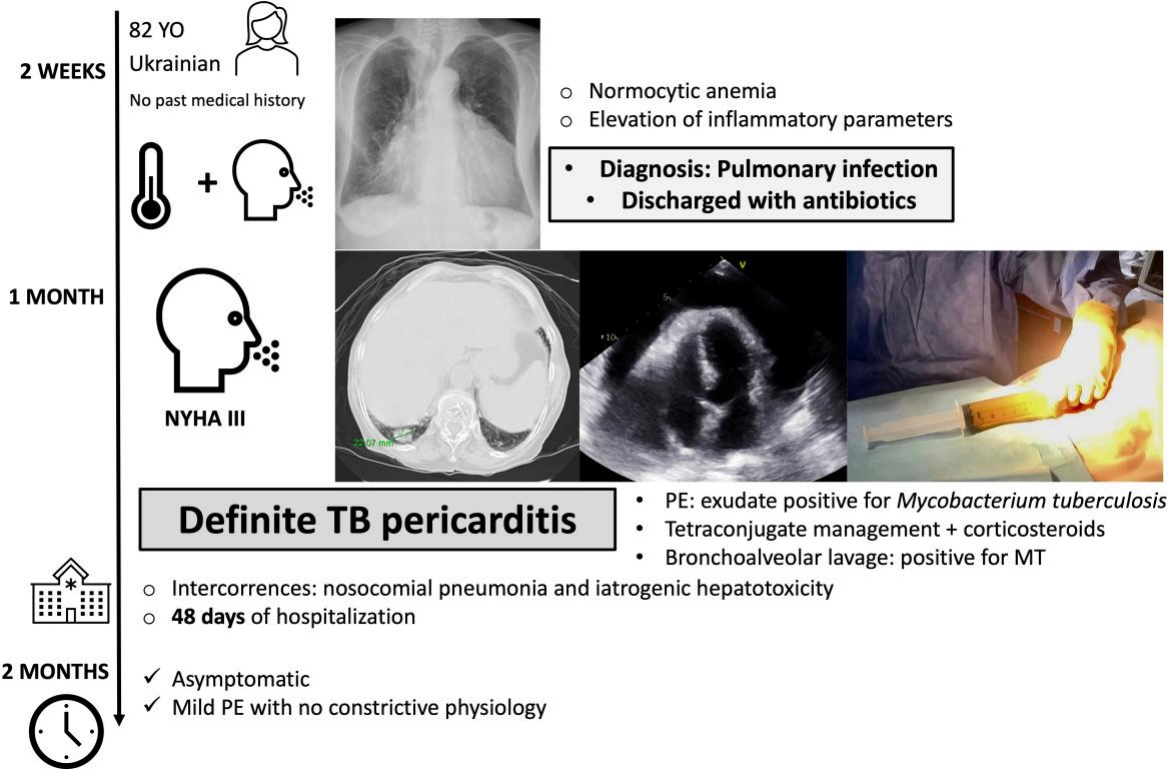


## Case presentation

An 82-year-old Ukrainian woman presented to the emergency department due to fever, dry cough, weakness, and anorexia for 2 weeks. She denied weight loss or night sweats and had no relevant medical history or medication. Physical examination revealed a temperature of 38.3°C, blood pressure of 105/71 mmHg, a heart rate of 99 b.p.m., and oxygen saturation of 95%. Cardiac auscultation indicated muffled heart sounds, while pulmonary auscultation was normal.

**Figure 1:**
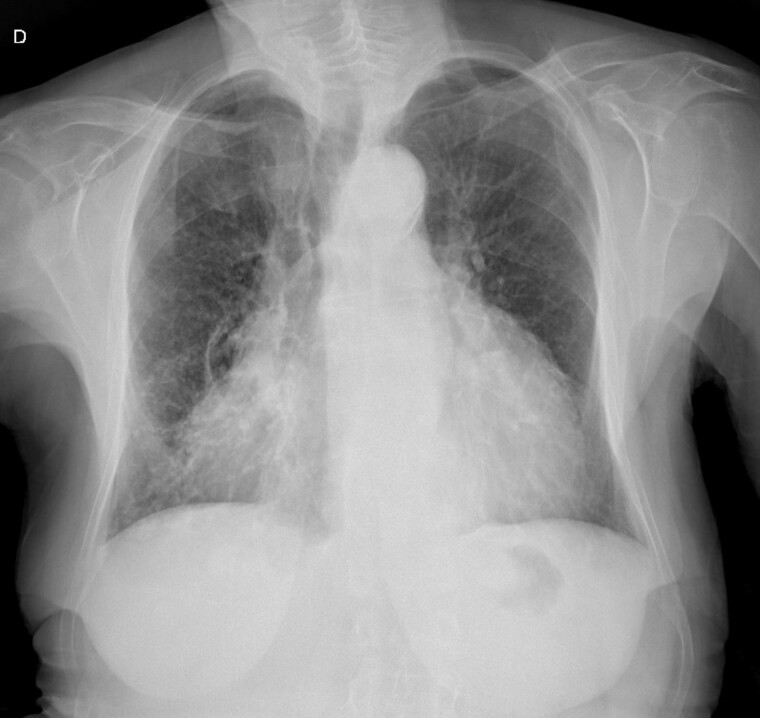
Initial chest X-ray.

**Figure 2:**
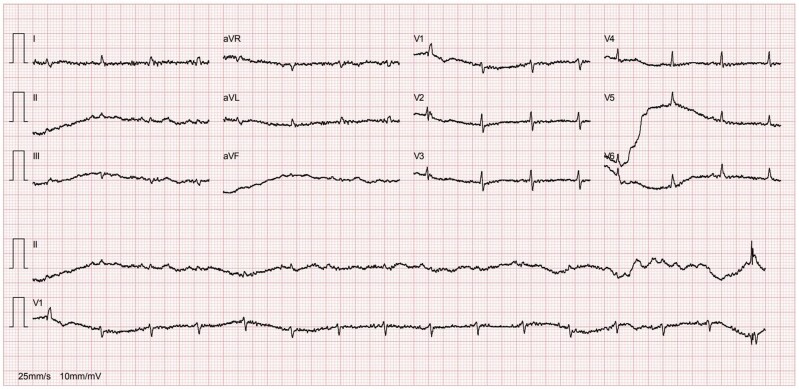
Electrocardiogram.

A chest X-ray (*Figure 1*) showed accentuation of the reticular pulmonary pattern, predominantly on the right base, and increased cardiothoracic index (CTI). The electrocardiogram presented a sinus rhythm, low voltage, and flattened T waves (*Figure 2*). The laboratory data revealed normocytic anaemia (10.8 g/dL, reference value 11.5–16 g/dL; mean corpuscular volume 87.3 fL, reference value 80–100fL) and a slight elevation of inflammatory parameters (C-reactive protein 57.6 mg/L, normal value ≤ 5 mg/L). A diagnosis of pneumonia was assumed, and the patient was discharged with antibiotherapy.

**Figure 3:**
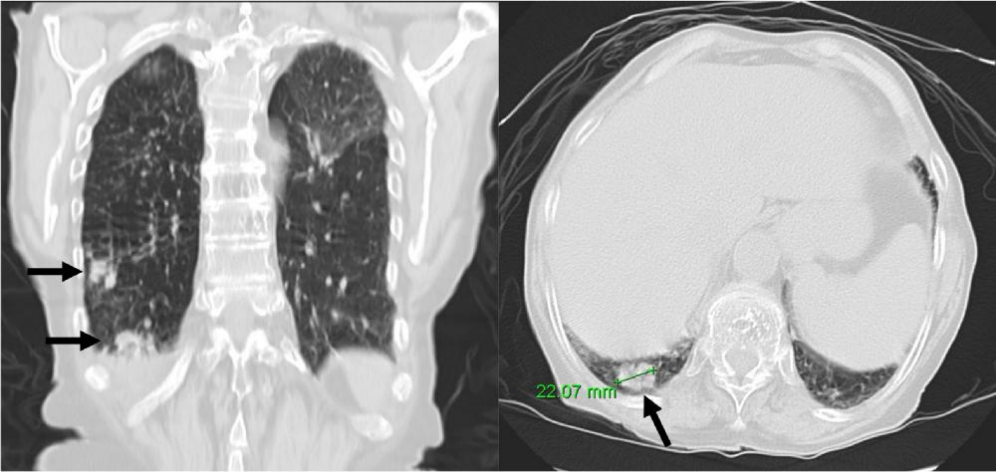
Nodular formations on thoracic computed tomography (arrows).

**Figure 4:**
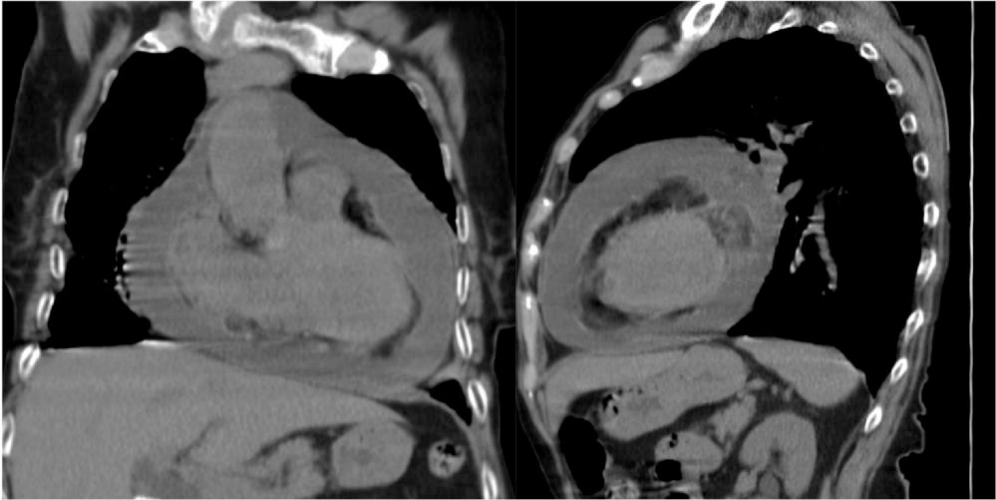
Pericardial effusion (thoracic computed tomography).

**Figure 5:**
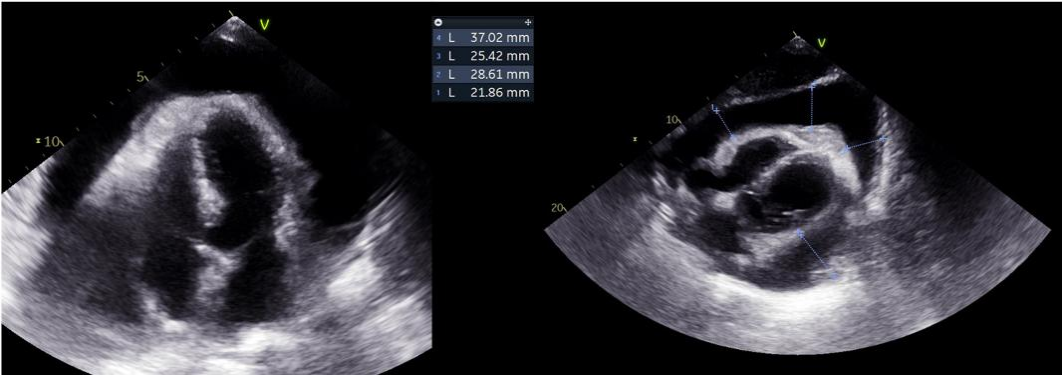
Pericardial effusion (transthoracic echocardiogram).

At 1-month follow-up, the patient had no fever but felt tired after mild exertion, corresponding to New York Heart Association Class III. The chest X-ray showed a greater CTI. A thoracic computed tomography described micronodular opacities on the right pulmonary base and two nodular formations, the larger measuring 22 mm (*Figure 3*). A large circumferential PE and thickened leaflets were also noted (*Figure 4*). A transthoracic echocardiogram (TTE) indicated large circumferential PE (*Figure 5*), with a maximum of 37 mm adjacent to the left heart cavities, showing a ‘swinging heart’ and echocardiographic signs of haemodynamic compromise (significant respiratory variation in transvalvular flow velocities and collapse of the right cavities). High-sensitivity cardiac troponins were negative (4 pg/mL, normal value < 10.8 pg/mL).

Emergency pericardial drainage via pericardiocentesis was performed, leading to active drainage of 1000 mL of homogeneous cloudy yellow PE. The control TTE revealed a thin slice of PE. Routine results of pericardial fluid were as follows: albumin 26.5 g/L, total erythrocyte count 5674/mm^2^, total nucleated cells count 1156/mm^2^ with 74% of polymorphonucleated cells, and lactate dehydrogenase 1599 U/L, with a pericardial fluid/serum ratio of 9.6 suggestive of exudate (>0.6). Adenosine deaminase (ADA) dosing was not performed, nor was the interferon-γ test. Aerobic, anaerobic, and mycologic cultures were negative. Acid-fast bacilli were not observed by the Ziehl–Neelsen method; however, the mycologic culture was positive, and PCR allowed the identification of *Mycobacterium tuberculosis* (MT). Mutations in antituberculosis drug-resistance-associated genes were not identified. Complementary laboratory studies, including thyroid hormone levels (thyroid stimulating hormone 0.4 µUI/mL, reference value 0.34–5.6 µUI/mL), viral serologies (including HIV), and immunologic analysis were normal.

Antituberculosis treatment was initiated with rifampicin 450 mg o.d., isoniazid 300 mg o.d., ethambutol 1000 mg o.d., and pyrazinamide 1500 mg o.d., along with dexamethasone 10 mg o.d., colchicine 0.5 mg o.d., and anti-inflammatory medication. A bronchoalveolar lavage confirmed the presence of MT in the tracheobronchial tree. Endobronchial biopsies revealed lymphocytic inflammatory infiltrate involving accumulations of epithelioid granulomas and some histiocytic cells.

The pericardial drainage tube was removed 4 days after pericardiocentesis, following more than 24 h without passive drainage. The hospitalization was complicated by nosocomial pneumonia and iatrogenic hepatotoxicity. The PE remained mild and stable. The patient was discharged after 48 days and referred to a pneumological diagnostic centre. The follow-up TTE showed a mild PE with no constrictive physiology, and the patient was asymptomatic.

## Discussion

*Mycobacterium tuberculosis* dissemination to the pericardium may occur through lymphatic spread, direct spread from the lungs, pleura^1,2^ or spine,^1^ or haematogenous spread.^1,2^

Four pathological phases of TB pericarditis are described: Stage 1/‘dry stage’, presenting with pleuritic chest pain, pericardial friction rub, and widespread ST elevation; Stage 2/‘effusive stage’, presenting with moderate/large PE^1^—bloody in 80%^6^—with or without CP; Stage 3/‘adsorptive stage’, presenting with CP and thick fibrinous pericardium fluid; and Stage 4/‘constrictive stage’ without residual fluid.^1^

This case describes a Stage 2 TB pericarditis in a patient from Ukraine who had recently travelled to Portugal. Ukraine falls into the upper–moderate incidence category.^3^ Current social and political issues may be contributing to the resurgence of diseases in low-incidence countries.

The typical presentation of acute pericarditis is rare in TB pericarditis,^6^ and clinical symptoms may vary according to the rate of pericardial fluid accumulation.^2^ Up to 50% of untreated cases progress to cardiac tamponade, and mortality rates can reach up to 85% at 6 months.^2^

The diagnosis of TB pericarditis may be challenging.^1,6,8^ Although limited, ADA is the most used biochemical test, and fluid microscopy and cultures are time-consuming.^1^ When the pericardium bacillary load is high, MT cultures and PCR techniques are frequently positive, which contrasts with paucibacillary condition that often result in negative tests.^9^ Our patient presented clinical and imagological findings of acute pericarditis, which, along with PCR identification of MT in the pericardial fluid, led to the diagnosis of acute TB pericarditis.

Empirical treatment in endemic areas is recommended for patients with exudative PE without other causes. However, this is not recommended for patients without a definitive diagnosis in non-endemic countries.^5,6^ In our patient, a definitive diagnosis was made after the identification of MT in the pericardial fluid, further confirmed in the tracheobronchial tree.

The objectives of the treatment are to control MT infection, relieve cardiac compression, and prevent complications.^1,8^ The use of corticosteroids in patients without HIV infection reduced the incidence of constriction and hospitalizations.^5,9^ Pericardiocentesis may also be associated with a lower risk of CP.^1,8^

Finally, if the patient fails to show clinical improvement or deteriorates after 4–8 weeks of medical therapy, pericardiectomy is recommended.^5^ This was not the case for our patient who remained clinically and echocardiographically stable. Multimodal imaging, including cardiac magnetic resonance, may help in diagnosing CP, with pericardial thickening over 6 mm being highly specific.^8^

## Conclusion

This case describes a definitive TB pericarditis in a non-endemic country. Awareness should be raised among clinicians of a potential increase in TB cases in non-endemic countries, especially considering increased immigration.

## Data Availability

The data underlying this article will be shared on reasonable request to the corresponding author.

## Lead author biography

Carolina Miguel Gonçalves was born on 20 December 1995 and holds a Master’s degree in Medicine from University of Coimbra. She is currently working as a cardiology resident at ‘ULS Região de Leiria’ in Portugal.

**Consent:** The authors confirm that written consent for submission and publication of this case report has been obtained from the patient in line with the COPE guidelines.

**Funding:** No funding was received for this work.
